# Common Error Pathways in CyberKnife™ Radiation Therapy

**DOI:** 10.3389/fonc.2020.01077

**Published:** 2020-07-08

**Authors:** Brandon T. Mullins, Lukasz Mazur, Michael Dance, Ross McGurk, Eric Schreiber, Lawrence B. Marks, Colette J. Shen, Michael V. Lawrence, Bhishamjit S. Chera

**Affiliations:** ^1^Department of Radiation Oncology, University of North Carolina Hospitals, Chapel Hill, NC, United States; ^2^Division of Healthcare Engineering, Department of Radiation Oncology, University of North Carolina School of Medicine, Chapel Hill, NC, United States; ^3^Carolina Health Informatics Program, School of Information and Library Science, University of North Carolina, Chapel Hill, NC, United States

**Keywords:** CyberKnife, common error pathways, SBRT, SRS, safety, radiation therapy

## Abstract

**Purpose/Objectives:** Stereotactic radiosurgery (SRS) and stereotactic body radiation therapy (SBRT) may be considered “high risk” due to the high doses per fraction. We analyzed CyberKnife™ (CK) SRS and SBRT-related incidents that were prospectively reported to our in-house incident learning system (ILS) in order to identify severity, contributing factors, and common error pathways.

**Material and Methods:** From 2012 to 2019, 221 reported incidents related to the 4,569 CK fractions delivered (5.8%) were prospectively analyzed by our multi-professional Quality and Safety Committee with regard to severity, contributing factors, as well as the location where the incident occurred (*tripped*), where it was discovered (*caught*), and the safety barriers that were traversed *(crossed)* on the CK process map. Based on the particular step in the process map that incidents *tripped*, we categorized incidents into general error pathways.

**Results:** There were 205 severity grade 1–2 (did not reach patient or no clinical impact), 11 grade 3 (clinical impact unlikely), 5 grade 4 (altered the intended treatment), and 0 grade 5–6 (life-threatening or death) incidents, with human performance being the most common contributing factor (79% of incidents). Incidents most commonly *tripped* near the time when the practitioner requested CK simulation (e.g., pre-CK simulation fiducial marker placement) and most commonly *caught* during the physics pre-treatment checklist. The four general error pathways included pre-authorization, billing, and scheduling issues (*n*= 119); plan quality (*n*= 30); administration of IV contrast during simulation or pre-medications during treatment (*n*= 22); and image guidance (*n*= 12).

**Conclusion:** Most CK incidents led to little or no patient harm and most were related to billing and scheduling issues. Suboptimal human performance appeared to be the most common contributing factor to CK incidents. Additional study is warranted to develop and share best practices to reduce incidents to further improve patient safety.

## Introduction

Stereotactic radiosurgery (SRS) and stereotactic body radiation therapy (SBRT) are effective treatments for primary and metastatic tumors in intracranial and extra-cranial disease sites (1–8). With growing clinical experience and technical advances, it is easy to assume that these modalities are performed with minimal risk of incidents. However, with any evolving technology, errors and incidents can occur; highlighted by incidents in France and the United States in the mid- to late-2000s (9–12). Thus, it is important to have reliable working technology, meticulous procedures, and robust quality assurance protocols (QA) to reduce the likelihood of errors and incidents ensuring safe utilization of the treatment modality.

A key component of quality and safety in radiation oncology is incident reporting and learning (13–16). Incident learning systems (ILS) are valuable in elucidating both random and systematic errors as they foster reporting of deviations, near-misses, and incidents (13, 17, 18). Effective learning systems also provide analysis of contributing factors leading to incidents (13). In our department, we utilize a robust ILS termed the “Good Catch Program” (19) to systematically report and analyze safety incidents. The reporting system is available to all staff to report any quality or safety concern in real time (i.e., something that reached the patient; a near miss; or an unsafe condition). Analysis of each report (where possible/appropriate) yields an incident description, how the incident occurred, how it was discovered, and the corrective response. Given the robustness of the system, and positive patient safety culture within the department, a substantial number of the quality or safety concerns are reported and analyzed (19). The accumulating data provide a strong source of information on common errors within different radiation treatment modalities.

Given the relatively “higher risk” nature of SRS/SBRT, due to the high doses per fraction and limited number of delivered fractions (i.e., less room for error), and paucity of reporting of common safety events in the literature, sharing our incident/event experience with delivering Cyberknife (CK) SRS/SBRT is felt to be beneficial. Thus, we herein report an analysis of CK SRS/SBRT-related incidents prospectively reported to our in-house ILS in order to identify common potential error pathways.

## Materials/Methods

A process map outlining the steps for the course of patients receiving CK, from pre-patient visit (prior to initial consult) to treatment delivery, was created. A multidisciplinary team including physicians, physicists, dosimetrists, radiation therapists, nurses, administrators, and members of our healthcare engineering division were all involved in our quality improvement and patient safety efforts. A process map for external beam radiation therapy (EBRT) was already present in the department and provided a source for comparison.

CK SRS and SBRT-related incidents that were previously prospectively reported to our in-house ILS from 2012 to 2019 were then reviewed. Our multi-professional Quality and Safety Committee, which meets weekly to review and analyze each of the submitted reports, initially analyzed these incidents. The committee is led by a physician and typically consists of multiple physicians, medical dosimetrists, medical physicists, radiation therapists, nursing personnel, and administrative staff to obtain substantial multi-disciplinary input. Each incident is evaluated with regard to severity, contributing factors, the location of where the incident was *tripped* (generated) and *caught* (discovered) on the CK process map, and the safety barriers that were traversed. Severity is rated from 1 through 6: 1—no patient impact, did not reach patient; 2—mild, no direct clinical impact; 3—moderate, clinical impact unlikely; 4—severe, altered the intended treatment; 5—life-threatening; and 6—death.

Based on the particular process step in the process map that incidents *tripped*, we categorized incidents into general error pathways (types) based on commonality. For example, incidents in which there were issues with target identification, target localization inaccuracies, or hardware/software issues leading to planning errors were categorized into “problem with poor plan quality” given that they could all lead to a treatment plan with a significant problem. Distribution diagrams were created for each general error type illustrating the breakdown of incidents.

## Results

**Figure 1:**
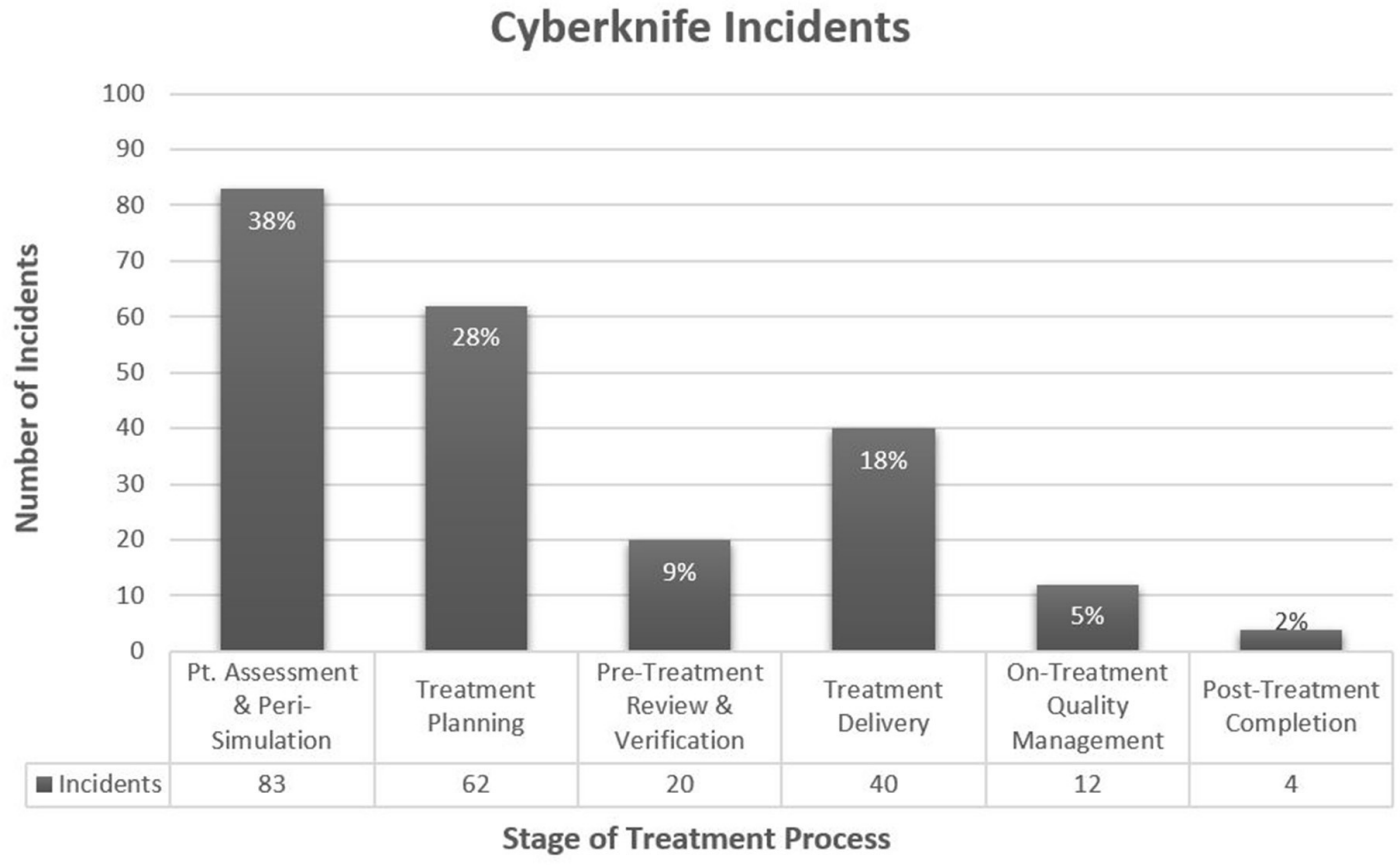
CyberKnife Incidents by Region of Treatment Process. *Percentages are of 221 incidents. Peri-Simulation includes steps related to the scheduling of simulation and special procedures (e.g., Fiducial placement, additional CK-associated imaging, etc.), and the actual simulation.

From 2012 to 2019, there were 221 reported incidents related to the 4,569 CK fractions delivered (5.8% rate). The CK process map consisted of 155 steps with 88 of those being safety barriers (compared to 158 and 94, respectively in the EBRT process map; see Appendix). Of the 221 reported incidents, a *tripped* and *caught* step along the CK process map could be ascribed to 184 (83%) and 160 (72%) incidents, respectively. The remaining incidents *tripped* or *caught* outside the analyzed process map (e.g., originating in other departments, ending by unplanned MD/nurse chart review, related to patient phone calls, etc.). Most incidents *tripped* around consultation and simulation (Figure 1), with the majority occurring around the time of the physician (attending or resident) requesting CK CT-simulation (CT-sim) (54 incidents, 24%). These incidents frequently involved issues with obtaining associated pre-CK CT-sim diagnostic imaging, fiducial marker placement, and/or insurance prior authorization. Incidents second most commonly *tripped* around the time of CK treatment planning (Figure 1).

Amongst the professional groups, nurses, physicians (including attending and residents), and physicists/dosimetrists reported the most incidents with 60 (27%), 57 (26%), and 37 (17%) reports, respectively (Table 1). Physicists and dosimetrists were combined into one category given that physicists do the majority of the CK planning with dosimetrists assisting as needed based on workload. Most common contributing factors to the incidents included performance (79% of incidents), communication breakdown (26%), environmental/technical factors (25%), and lack of specific policies/procedures (17%) (Table 1).

**Table 1:** Distribution of CK incidents.

	**CK incidents *N* = 221**
**Incident categories**	
Scheduling	104 (47%)
Plan quality	30 (14%)
Issues with pre-meds, IV contrast, consent	25 (11%)
Prior authorization/billing	15 (7%)
Image guidance	12 (5%)
Record and verify dose recording/tracking	11 (5%)
Other	24 (11%)
**Professional group reporting incident**	
Nurses	60 (27%)
Physicians	57 (26%)
Physicists/dosimetrists	47 (22%)
Radiation therapists	34 (15%)
Administration/clerical staff	23 (10%)
**Contributing factors**	
Performance	175 (79%)
Communication	52 (26%)
Environmental/technical factors	55 (25%)
Policy or procedure not being in place	38 (17%)
Workload	36 (16%)
Cross coverage/handoffs	8 (4%)
**Incident severity**	
1 (No patient impact)	115 (52%)
2 (Mild)	90 (41%)
3 (Moderate)	11 (5%)
4 (Severe)	5 (2%)
5 (Life-threatening)	0 (0%)
**Incidents reaching patient**	
Yes	106 (48%)
No	115 (52%)

*Physics and Dosimetry are grouped together in the professional group distribution given that the majority of our CK planning is done by Physics within our workflow. Pre-Meds are medications provided prior to CK treatment (i.e. steroids)*.

Barriers that most commonly *caught* incidents included the physics pre-treatment chart check (14 incidents *caught*, 9% of all incidents), our daily nursing on-treatment assessment visits (11 incidents, 7% of all incidents), our daily/weekly physician on-treatment management visits (9 incidents *caught*, 6% of all incidents), and our daily simulation review (7 incidents *caught*, 4% of all incidents). Common traversed safety barriers included radiation therapist verification that patients had on-treatment visits scheduled appropriately and CT-sim therapists verifying the simulation directive. By professional role, incidents were most commonly *tripped* by physicians (attending and residents) and therapists, most commonly *caught* by physicians and nurses, with most commonly traversed barriers associated with physicians and physicists.

The four common general error pathways included pre-authorization, billing, and scheduling issues (*n* = 119); plan quality (*n* = 30); administration of IV contrast during simulation or pre-medications during treatment (*n* = 22); and image guidance (*n* = 12). Of the 221 incidents, 183 (83%) fell into one of these four error groups. Table 2 demonstrates the distribution of incidents for each of the four common error pathways. Of the 30 incidents related to poor plan quality, 29 were detected prior to treatment. The other incident was detected after treatment was completed, but led to no adverse patient effect. All image guidance-related incidents (12) were detected with correction prior to treatment.

**Table 2:** Distribution of incidents for common general error pathways.

**Common General Error Pathways**	**Number of CK Incidents**	**Severity Range**
**A. Prior-authorization, billing, and scheduling problems** • Delay in CK-Tx associated consults, imaging, or fiducial placement ° Delay in consults for quality assurance (e.g. neurosurgery) ° Delay in imaging needed for target delineation ° Delay in fiducials needed for CK localization • Patient scheduling delays/errors • Prior authorization/billing issues ° Late prior-authorization obtained ° Fractionation change during planning without billing team notification leading to late prior-authorization	119 • 12 ° 3 ° 6 ° 3 • 92 • 15 ° 5 ° 10	1–4 • 1–4 ° 1–2 ° 2–4 ° 2–3 • 1–4 • 1–2 ° 1–2 ° 1–2
**B. Problem with poor plan quality** • Issue with target identification or dosing pattern prescribed ° Target identification error ° Dose and fractionation pattern incorrect ▪ Physician error or changed mind ▪ Dose/fx and fx number reversed ▪ Dose/fx in prescription and plan differ, reason unclear ° Previous treatment not considered • Tracking issue • Planning Error ° Hardware/Software issues ▪ Wrong collimator size used ▪ Inconsistencies between plan and prescriptions ° Contoured targets not included in plan ° OAR not spared ▪ OAR not contoured ▪ OAR laterality incorrect ▪ Planned on incorrect CT ° Margins not applied correctly	30 • 13 ° 2 ° 8 ▪ 6 ▪ 1 ▪ 1 ° 3 • 4 • 13 ° 5 ▪ 1 ▪ 4 ° 2 ° 4 ▪ 2 ▪ 1 ▪ 1 • 2	1–4 • 1–4 ° 1–4 ° 1–4 ▪ 1 ▪ 1 ▪ 4 ° 1–3 • 1–2 • 1–2 ° 1 ▪ 1 ▪ 1 ° 1 ° 1–4 ▪ 2–4 ▪ 1 ▪ 1 • 1
**C. Administration of IV contrast during CT-simulation or pre-medications during treatment** • Pre-meds not given when recommended or given inappropriately ° Inappropriate administration of pre-meds ° Pre-meds not provided though ordered ° Pre-meds indicated but not ordered • Conflicting Pre-med Instructions • IV Contrast Issues ° Conflicting instructions ° No creatinine check before administration • Delay in Pre-Med Administration Delaying Tx	22 • 7 ° 2 ° 2 ° 3 • 3 • 6 ° 5 ° 1 • 6	1–3 • 1–3 ° 2–3 ° 1–2 ° 1–3 • 1–2 • 1–2 ° 1–2 ° 1 • 1–2
**D. Issue with image guidance** • CT Simulation Inadequate ° 4D incorrectly not performed • Issue with 3D Dataset Used for Planning ° Poor image fusion ° Outdated fusion MRI ° Significant delay in image fusion	12 • 2 ° 2 • 10 ° 4 ° 3 ° 3	1–4 • 2 ° 2 • 1–4 ° 1–4 ° 1–2 ° 1

*Distribution of incidents for the common general error pathways for CK treatment. “Severity range” describes the range of incident severity per category. Fx, Fraction; Tx, Treatment; OAR, Organs at risk*.

The majority of incidents had no direct clinical impact; i.e. severity grade 1–2 (93%). There were 11 (5%) severity grade 3 incidents, 5 (2%) severity grade 4 incidents, and no severity grade 5 incidents. Forty eight percent of incidents reached the patient in some capacity. Despite many incidents not leading to patient consequences, some could have led to severe consequences if not prevented by safety barriers. Some selected notable grade 4 incidents within our generic error pathways include the following:

1. Patient receiving CK for a brain metastasis. Plan intent was 30 Gy in 5 fractions but was incorrectly calculated as a single 30 Gy fraction. Fortunately, incident was caught and corrected during physics pre-treatment chart check.
2. Patient receiving CK for an AVM and had multiple prior AVM EBRT and CK treatments. Consideration/planning for additional CK treatment was underway based on our understanding that the patient had received one prior CK treatment (as was documented in the record and verify system). Fortunately, we subsequently identified that the patient had actually received two prior CK treatments, and our planned treatments were modified accordingly.
3. Patient receiving CK for a right lower lobe lung lesion. Patient's liver was inadvertently not segmented as an organ-at-risk. During physics pre-treatment chart check, the reviewing physicist noticed this, and when subsequently segmented, the plan was failing our liver dose/volume constraint. The patient was re-planned and rescheduled without patient harm.
4. Patient who had prior thoracic spine radiation was being planned to receive CK for a thoracic spinal metastasis. Due apparently to a new vertebral fracture, the CT images from the prior and current plan were initially mis-registered to each other during planning. Fortunately, this was caught and corrected during physics pre-treatment check, the patient was re-planned, and there was no adverse patient impact. If uncorrected, the degree of overlap between the prior and current plan was greater than believed and the dose to the adjacent normal tissues would have been higher than considered safe. Now we always request DICOM images when possible to ensure accuracy of prior radiation treatments.

## Discussion

This analysis of reports to our departmental incident learning system demonstrates potential hazards and common error pathways for CK SRS/SBRT delivery. Given that our safety culture encourages incident reporting (20, 21), the presented incidents and error pathways, while not meant to be exhaustive, likely represent a sufficient sample of incidents to draw some general insights.

First, it was notable that there were less safety barriers in the CK process map compared to the EBRT process map, despite the increased complexity of CK treatment. This was mostly due to the lack of dosimetry involvement with pre-treatment chart checking as well as lack of a quality assurance (QA) day being present within the CK process map (compared to that of EBRT). Within EBRT, typically dosimetrists perform treatment planning and some initial quality assurance with their pre-treatment chart check. A second pre-treatment check is later performed by a physicist to ensure optimal safety. However, in CK, our physicists perform both the treatment planning and the initial quality assurance with their pre-treatment chart check. There is no safety barrier to provide a double check of the physics pre-treatment check. Given the complexity of CK treatment, and lower margin for error, this may be an area for improvement to ensure that adequate safety barriers are in place to prevent incidents. It would likely be beneficial to increase the amount of interdepartmental peer review involved with CK planning quality assurance to ensure optimal safety. Notably however, our department has had a dual layer peer review process in place since the 1980s for all EBRT plans. In addition to traditional peer review with chart rounds, we have an upstream pre-treatment peer review of treatment intent, contours, fields, etc. In 2011, we also included all CK plans in our dual peer review process serving as an extra upstream barrier against incidents.

Second, the methods of error generation with CK appear different compared to EBRT. With CK, extra-cranial targets (e.g., lung, liver, prostate, pancreatic, etc.) are usually implanted with internal fiducial markers prior to planning/treatment for localization (both pre- and during CK) to increase accuracy and safety. However, this additional process can lead to more incident generation as human or technical errors surrounding this process can cause potential targeting inaccuracies (four associated incidents; Table 2). In addition, our colleagues in other departments (e.g., radiology or urology) place most of our fiducial markers, thus requiring robust inter-departmental communication to ensure that procedures are performed as needed (e.g., within proper time windows, in desired locations) (three associated incidents; Table 2). Billing for CK is more complex than for EBRT, with prior authorization usually necessary. This can further lead to incidents with patient treatment delays (fifteen associated incidents; Table 2). Finally, we commonly administer pre-medications with CK treatments (e.g., steroids for brain and prostate) in order to help prevent acute side effects. This creates a new avenue for incident production potentially due to conflictions with pre-med instructions, inappropriate pre-medication administration, or delays in pre-medications delaying treatments (16 incidents; Table 2), all of which can potentially lead to adverse patient consequences.

Despite these differences, there were also some notable similarities between CK and EBRT incident generation. First, where incidents were *tripped* and *caught* in the CK process is similar. We observed that most CK incidents generate during pre-simulation, near the time of request of CK CT-sim, and during treatment planning. These results are also consistent with many reports with EBRT. The World Health Organization (WHO) reviewed over 4,500 near misses within radiation therapy and noted that a majority were associated with peri-simulation and initial planning (22). Many single institution studies also note similar distributions of pre-treatment incidents (19, 23–29). Second, the contributing factors to incident production were similar. Our analysis found that most (~80%) of incidents were attributed to performance issues (with the majority arising from human error), followed by communication breakdown, environmental/technical factors, and a lack of standardized processes in place. Multiple reports note a shift in incident generation and causation with the introduction of newer and more advanced technologies (29–34). When workers are learning to manage newer machines (e.g., CK or IMRT technology) and steps/policies are less standardized (or evolving with experience), increases in human error are common and can propagate (33). This high frequency of performance issues (e.g., not following standardized processes/procedures, suboptimal documentation, lack of teamwork, etc.) is consistent with multiple reports in the literature including from the WHO, NRC, and Radiation Oncology Safety Information System (12, 22, 35)^1^. It is difficult to determine how best to specifically improve these factors; however, ensuring proper personnel training, having standardized processes for documentation and reporting, using robust procedural checklists, and utilizing a thorough QA program are steps that can improve error rates.

Similar to other reports in the literature (12, 20, 22, 29, 34, 36, 37), procedural checklists were important in catching a large fraction of the incidents in our clinic (e.g., during routine pre-treatment QA checks via checklists, and especially, the physics pre-treatment chart check catching the most incidents). Over the years, our physics pre-treatment check has evolved to include an increasing number of elements aimed to catch incidents. Currently, it consists of 24 high-quality elements ranging from checking accuracy of the treatment prescription to ensuring completeness and accuracy of billing. Given the reliance we place on this checklist, it was reassuring to see its effectiveness in catching many incidents. Given that the majority of CK incidents occurred around simulation and treatment planning, adding and improving procedural checklists within these areas, and upstream, could be useful for incident reduction.

Limitations to our study include the single-institutional nature, and thus the reported incidents, barriers, and error pathways may not be representative of all institutions. However, our strong safety culture and robust incident reporting system aids in identifying many incidents that we suspect are likely occurring nationally. Additionally, the study is limited by the nature of incident reporting data, which is subject to reporting biases and may not detect all incidents.

Generally speaking, SBRT and SRS planning and treatment delivery is more complex than EBRT. In addition, with the use of only one or a few high fractional doses, an error will likely have more clinical implications than an error for a patient receiving more conventional fractionation. Fortunately, the reported incidents in our study had relatively low patient severity as the majority (93%) had no direct clinical impact, only 2% altered the intended treatment, and none were life-threatening. However, as demonstrated by the selected cases, some of these incidents could lead to severe consequences if not prevented. Thus, this highlights the importance of continual assessment of error pathways, and performance of safety barriers, to further reduce incidents and improve quality/safety.

In conclusion, our study demonstrates potential hazards and common error pathways related to CK SRS/SBRT. Given the risks associated with high fractional doses, continual attention to this issue is needed.

## Data Availability Statement

The datasets generated for this study are available on request to the corresponding author.

## Ethics Statement

This study involving human participants was reviewed and approved by the University of North Carolina Institutional Review Board. The participants provided their written informed consent to participate in this study.

## Author Contributions

BC, BM, and LM: conceptualization. BM, MD, RM, ES, CS, ML, and BC: data curation. BM, BC, LM, MD, and RM: formal analysis. BM and BC: statistical analysis. BM, LM, LBM, and BC: writing—original draft. All authors: writing—review and editing.

## Conflict of Interest

BC and LM have a financial relationship (e.g., royalties and equity) with CommunifyHealth, which provides software for incident reporting and analysis. None of the research presented in this manuscript has been spearheaded by this relationship. The remaining authors declare that the research was conducted in the absence of any commercial or financial relationships that could be construed as a potential conflict of interest.

## Footnotes

^1^*ROSIS: Radiation Oncology Safety Information System*. Available online at: https://roseis.estro.org/ (accessed October 18, 2019).
