# Supplementary material for: Common Error Pathways in CyberKnife™ Radiation Therapy
Source: Front Oncol. 2020 Jul 8;10:1077. doi: 10.3389/fonc.2020.01077 (PMC7360810; doi:10.3389/fonc.2020.01077)
Supplement: Supplementary file 1 [file Data_Sheet_1.PDF]

Appendix

A) EBRT Process Map

Pre-visit

- Admin registers Pt Demographics & insurance in EPIC and issues MRN
- Admin evalutes insurance eligibility for consult (MN ck if P&P exist)
- Admin schedules Pt consult and creates recurring accounts
- Admin enters Pt information into MQ
- Admin verifies receipt of Pt outside records
- Admin submits Pt outside records into EMRs and delivers to Radiology if needed

Pt Assessment

- Attending, Resident or APP reviews pertinent medical records (pathology report, imaging and outside records)
- Attending decides to treat and determines urgency as applicable
- Attending, Resident or APP discusses patient case at Tumor Board as applicable (evaluation of other Tx modalities: chemo, surgery)
- Pt checks in for consult
- Admin verifies Pt ID at check-in
- RN or CST takes Pt vital signs
- RN completes nursing assessment, inlcuding 3Ps
- RN provides Pt education
- RN communicates with ancillary services as applicable (social work, nutrition, speech, CSSP, pain team, smoking cessation etc.)
- RN documents nursing assessment in EPIC and MQ
- Attending, Resident or APP interviews, examines, assesses Pt, determines intent to treat
- Attending, Resident or APP consents Pt
- Attending, Resident or APP orders f/u appt, CT sim, labs, imaging, special procedures through Admin via Onc Flowsheet, email, call, or EPIC inbasket
- Attending, Resident or APP enters documentation, meaningful use, billing into EMR
- Attending reviews and approves documentation, meaningful use, billing entered into EMR
- Attending, Resident or APP defines and enters Dx and clinical staging into EMRs
- Select clinical protocol. Select clinical trial
- CST scans consent and exports to MQ
- Pt checks out at front desk (with AVS, if non-status check appt.)
- Admin schedules special procedures (fiducial or Calypso placement, etc.) and additional imaging as applicable
- Admin schedules Sim
- Admin reviews Sim schedule to identify authorization needs
- Non-RadOnc Provider performs special procedures as applicable
- Attending or Resident enters Sim Assessment (including ordering immobilization, 4D etc.)

Imaging for RT Planning

- CT-sim RTT verifies Sim Assessment
- CT-sim RTT verifies Sim Assessment approval by MD
- Pt checks in
- RN verifies request for IV contrast in MQ, if IV contrast applicable
- RN completes IV contrast screening form, if IV contrast applicable
- RN places PIV and draws labs if needed, if IV contrast applicable
- CST scans completed IV contrast screening form into MQ, if IV contrast applicable
- CST queues Pt to Sim, if IV contrast applicable
- CT-sim RTT verifies Pt ID
- CT-sim RTT constructs necessary immobilization and ancillary devices
- CT-sim RTT performs Surview scan
- Attending or Resident reviews and verifies Pt setup in room
- CT-sim RTT enters if contrast is used in CT console
- RN verifies blood return and flushes central line, if IV contrast and central line applicable
- CT-sim RTT administers contrast if needed
- CT-sim RTT acquires CT scan
- CT-sim RTT pages Attending and Resident (if absent)
- Attending or Resident places isocenter
- Attending reviews isocenter
- CT-sim RTT transfers images and isocenter coordinates to DICOM target
- CT-sim RTT marks, tattoos and photographs reference point on Pt and/or marks localization device
- CT-sim RTT schedules Pt Tx time
- CT-sim RTT orients and dismisses Pt (Pt orientation)
- CT-sim RTT schedules Pt status checks and hold spots on Tx machine
- CT-sim RTT completes RTT Sim Assessment Checklist
- CT-sim RTT messages via QCL task list: CMD (Tx plan, pacemaker, CT ready to import), MP (consult if needed), Research (Sx mgmt), IMRT pre-authorization, Sim Review, Chart Rounds
- MD enters Clinical Tx Plan note and Sim note
- Admin obtains pre-authorization codes from Clinical Tx Plan note and Sim Assessment
- Admin contacts insurance company to obtain authorization to treat Pt
- Admin documents authorization is complete in MQ and EPIC
- MP consult

Treatment Planning

- CMD transfers CT-sim data to TPS and creates MD Start Plan
- MD orders image fusion and image guidance via Assessment and QCL
- MP fuses image sets
- CMD/Attending or Resident draws OAR contours
- MD draws target contours
- Attending reviews OAR and target contours
- Attending or Resident writes Tx planning note in MQ: Tx site, total dose, fxs, Tx technique, bolus, laterality
- Attending reviews Tx plan note
- Attending, Resident, MP, CMD, RTT, RN, Admin, Students review contours and beam geometry at Sim review meeting
- CMD messages suggested changes to Attendings
- Resident documents Sim review QCL is complete
- Attending changes contours if needed
- CMD pre-plans: prioritizes workload for IMRT QA day via QCL
- CMD pre-plans: compare Tx plan note from all sources and confirm changes if needed
- CMD pre-plans: compare image label vs. image appearance
- CMD pre-plans: verify contours were reviewed by Attending after Sim review
- CMD calculates and optimizes dose distribution
- CMD checks plan parameters via Raystation scripts and DOS pre-Tx checklist
- MP checks VMAT/IMRT plan pre-Attending
- CMD messages Attending and Resident for plan review
- Attending reviews/approves plan in TPS
- CMD transfers plan from TPS to MQ
- Attending approves Rx and other documents (goalsheet) in MQ
- CMD enters chart parameters (confirm tol tables, site setup, create tx calendar) + image documentation.
- CMD completes DOS pre-Tx check
- CMD schedules pre-Tx check with MP via QCL

Pretreatment review and verification

- MP enters CBCT and VisionRT prep as applicable
- MP reviews plan in TPS
- MP completes PHY pre-Tx check via PHY pre-Tx checklist
- MP schedules chart write-up with RTT via QCL
- CT-sim RTT enters chart write-up
- CT-sim RTT compares Tx plan documents to Tx delivery documents
- CT-sim RTT reconciles messaging via QCL and schedule with Tx plan
- CT-sim RTT checks for completion of PHY pre-Tx check
- CT-sim RTT completes and approves chart write-up
- CT-sim RTT adds ports/CBCT to Tx calendar and manually enters MUs as applicable
- RTT verifies Pt addition to VisionRT
- RTT verifies IMRT QA completed as applicable
- RTT verifies IMRT pre-authorization is complete
- RTT retrieves Pt and identifies them by 2 independent means.
- RTT prepares Tx table according to setup notes
- RTT introduces procedure to Pt
- Pt confirms ID on monitor in maze
- RTT sets up Pt on Tx table according to setup notes
- RTT assesses Tx field visually on Pt and messages MD as applicable
- RTT performs verbal timeout with partner via MQ Assessment
- RTT images Pt (port, CBCT, VisionRT) until within MD's acceptable parameters
- RTT captures couch coords (eChart/Note docum by 2 RTTs), as applicable. Insert Couch Capture note
- RTT chooses setup image, via marking as "QA DAY", "QA FILM", "DIBH", etc
- RTT records SSDs, via eChart/Notes
- RTT marks isocenter or a visible field on Pt, via sharpie
- Pt leaves vault
- MD reviews and approves QA Day onboard imaging; orders shift if needed

Treatment delivery

- RTT performs daily AM QA on linac & imagers; at clinic open time
- RTT enters QA results, via Atlas; at clinic open time
- RTT messages POD if daily AM QA fails; at clinic open time
- RTT verifies Pt IP/OP status, via comparision of Epic to RTT memory; at clinic open time
- RTT obtains Attending MD approval if Pt IP/OP status changed, as applicable
- RTT communicates change of Pt IP/OP status to Admin, as applicable
- Admin flags in EPIC if Pt changes from OP to IP, as applicable
- EPIC transfers IP account to MQ, as applicable
- Admin creates Pt OP account in EPIC if Pt changes from IP to OP, as applicable
- Admin verifies Pt OP status by unchecking IP checkbox in MQ if Pt changes from IP to OP, as applicable
- RTT views Mq Tx record
- RTT schedules IP Pt transportation (at clinic open time) or contacts OP Pt
- RTT perform site setup, per site, verifies setup via self-check, approves setup in Mq
- RTT walks Pt from lobby to vault
- RTT verifies Pt ID by 2 independent means
- Pt confirms ID on monitor in maze
- RTT performs Pt site setup, according to setup notes (immobilization devices, shifts), per site
- RTT visualizes anterior light fd on Pt, per site; redraws isocenter or visible field on Pt, if needed
- RTT compares R&V setup (clinical parameters, Tx consent) vs. actual, with RTT partner via time-out
- RTT documents Tx setup details & RTT partner time-out agreement
- RTT images Pt per Attending's orders
- MD reviews & approves setup image; orders shifts if needed
- RTT re-couch-copies table parameters if MD orders shifts from previous setup image
- RTT deliver Tx per Attending's Rx
- RTT observes Pt on monitors for duration of Tx
- RTT records Tx, if CK
- Mq records Tx, if not CK
- Repeat from "RTT performs Pt site setup", if untreated sites remain
- RTT captures charge if Tx sucessful
- RTT queues Pt to next location, if Mq prompts that appt's remain
- Pt leaves vault
- RN or CST takes vital signs, if status check day
- RN completes nursing assessment, if status check day
- RN communicates with ancillary services as applicable, if status check day
- RN documents assessment in EPIC, if status check day
- RN queues Pt to Provider via page or verbal communication, if status check day
- RTT messages Attending for final Tx now, via Resident, if final Tx
- RTT marks Pt as completed & inactive, if final Tx
- MD orders post-Tx appointments, labs, imaging, special procedures from Admin via Onc Flowsheet, email, call, EPIC inbasket, if last status-check

On-Tx quality management

- PHY completes weekly physics chart check, including Continuing Physics charge captures
- MD, RN, Ancillary Services completes weekly management visit, social work, and nutrition
- MD, PHY, DOX, RTT complete weekly Chart Rounds via review of the following: dose distribution (DVHs, isodoses); Rx (dose, dise/Fx, total dose); dose site summary; goal sheets; port films; plan parameters; delivery; Tx Planning note, Dx code
- Admin reviews charges daily

Post-Tx completion

- Pt carries Yellow Sheet to Front Desk to trigger post-tx Appt scheduling
- Admin schedules post-Tx appointments using information from Onc Flowsheet

B) CK Process Map

Pre-visit

- Admin registers Pt Demographics & insurance in EPIC and issues MRN
- Admin evalutes insurance eligibility for consult (MN ck if P&P exist)
- Admin schedules Pt consult and creates recurring accounts
- Admin enters Pt information into MQ
- Admin verifies receipt of Pt outside records
- Admin submits Pt outside records into EMRs and delivers to Radiology if needed

Pt Assessment

- Attending, Resident or APP reviews pertinent medical records (pathology report, imaging and outside records) -
- Attending decides to treat and determines urgency as applicable
- Attending, Resident or APP discusses patient case at Tumor Board as applicable (evaluation of other Tx modalities: chemo, surgery)
- Pt checks in for consult
- Admin verifies Pt ID at check-in
- RN or CST takes Pt vital signs
- RN completes nursing assessment, including 3Ps
- RN provides Pt education
- RN communicates with ancillary services as applicable (social work, nutrition, speech, CSSP, pain team, smoking cessation etc.)
- RN documents nursing assessment in EPIC and MQ
- Attending, Resident or APP interviews, examines, assesses Pt, determines intent to treat
- Attending, Resident or APP consents Pt
- Attending, Resident or APP orders f/u appt, CT sim, labs, imaging, special procedures
- Attending, Resident submits SBRT referral form to Admin
- Attending, Resident or APP enters documentation, meaningful use, billing into EMR
- Attending reviews and approves documentation, meaningful use, billing entered into EMR
- Attending, Resident or APP defines and enters Dx and clinical staging into EMRs
- Select clinical protocol. Select clinical trial
- CST scans consent and exports to MQ
- Pt checks out at front desk (with AVS, if non-status check appt.)
- Admin schedules special procedures (fiducial or Calypso placement, etc.) and additional imaging as applicable
- SBRT Admin schedules Sim
- SBRT Admin reviews Sim schedule to identify authorization needs (seeks authorization)
- Non-RadOnc Provider performs special procedures as applicable
- Attending or Resident enters Sim Assessment (including ordering immobilization, confirming 3 Ps, 4D etc.)

Imaging for RT Planning

- CT-sim RTT verifies Sim Assessment
- CT-sim RTT verifies Sim Assessment approval by MD
- Pt checks in
- RN verifies request for IV contrast in MQ, if IV contrast applicable
- RN completes IV contrast screening form, if IV contrast applicable
- RN places PIV and draws labs if needed, if IV contrast applicable
- RN checks labs to ensure IV contrast can be administered if applicable
- Attending or Resident confirms contrast can be administered if IV contrast applicable
- CST scans completed IV contrast screening form into MQ, if IV contrast applicable
- CST queues Pt to Sim, if IV contrast applicable
- CT-sim RTT verifies Pt ID
- CT-sim RTT constructs necessary immobilization and ancillary devices
- CT-sim RTT performs Surview scan
- Attending, Resident or MP reviews and verifies Pt setup in room
- CT-sim RTT enters if contrast is used in CT console
- RN verifies blood return and flushes central line, if IV contrast and central line applicable
- CT-sim RTT administers contrast if needed
- CT-sim RTT acquires CT scan
- CT-sim RTT pages Attending and Resident (if absent)
- Attending reviews CT suitability for planning
- MP consult
- PHY reviews CT suitability for planning
- CT-sim RTT transfers images and isocenter coordinates to DICOM target
- CT-sim RTT adds simulation note detailing Pt setup including immobilization devices/patient orientation
- CT-sim RTT schedules Pt Tx time
- CT-sim RTT orients and dismisses Pt (Pt orientation)
- CT-sim RTT schedules Pt status checks and hold spots on Tx machine
- CT-sim RTT completes RTT Sim Assessment Checklist
- CT-sim RTT messages via QCL task list: PHY (Tx plan, pacemaker, CT ready to import), Research (Sx mgmt), SBRT pre-authorization, Sim Review, Chart Rounds
- MD enters Clinical Tx Plan note and Sim note
- SBRT Admin obtains pre-authorization codes (on day of Sim) from Clinical Tx Plan note and Sim Assessment
- SBRT Admin contacts insurance company to obtain authorization to treat Pt
- SBRT Admin documents authorization is complete in MQ and EPIC

Treatment Planning

- PHY transfers CT-sim data to TPS
- MD orders image fusion and image guidance via Assessment and QCL
- PHY fuses image sets, contours, and creates plan for MD contouring
- PHY/Attending or Resident draws OAR contours
- Resident draws target contours
- Attending reviews OAR and target contours
- Attending or Resident writes Tx planning note in MQ: Tx site, total dose, fxs, Tx technique, bolus, laterality
- Attending reviews Tx plan note
- Attending, Resident, PHY, CMD, RTT, RN, Admin, Students review contours at Sim review meeting, Resident completes Sim Review QCL item
- CMD/PHY messages suggested changes to Attendings
- Resident documents Sim review QCL complete
- Attending changes contours if needed
- PHY pre-plans: compare Tx plan note from all sources and confirm changes if needed
- PHY pre-plans: compare image label vs. image appearance
- PHY pre-plans: verify contours were reviewed by Attending after Sim review
- PHY calculates and optimizes dose distribution
- PHY messages Attending and Resident for plan review
- PHY completes plan "Time-Out" with the Attending approving plan
- Attending reviews/approves plan in TPS
- PHY schedules Plan Review QCL
- PHY transfers plan from TPS to MQ
- Attending approves Rx and other documents (goalsheet) in MQ
- PHY enters chart parameters (confirm tol tables, site setup, create tx calendar) + image documentation.
- PHY schedules pre-Tx check with PHY via QCL
- PHY schedules CK QA via QCL

Pretreatment review and verification

- Attending, Resident, PHY, CMD, RTT, RN, Admin, Students reviews Plan at Sim review meeting, Resident completes Plan Review QCL item
- PHY reviews plan in TPS
- PHY completes PHY pre-Tx check via PHY pre-Tx checklist
- PHY performs CK QA
- RTT verifies CK QA completed as applicable
- RTT verifies CK pre-authorization is complete
- RTT verifies patient has MD status checks scheduled prior to each treatment
- RTT retrieves Pt and identifies them by 2 independent means.
- RTT prepares Tx table according to setup notes
- RTT introduces procedure to Pt
- Pt confirms ID on monitor in maze
- RTT sets up Pt on Tx table according to setup notes
- RTT performs verbal timeout with partner via MQ Assessment
- RTT images Pt (port, CBCT, VisionRT)
- MD and PHYS verify patient setup via imaging
- Pt leaves vault
- MD reviews and approves onboard imaging; orders shift if needed
- MD enters therapy plan in Epic with pre-meds if applicable

Treatment delivery

- RTT performs daily AM QA on linac & imagers; at clinic open time
- RTT enters QA results, via Atlas; at clinic open time
- RTT messages PHY if daily AM QA fails; at clinic open time
- RTT verifies Pt IP/OP status, via comparision of Epic to RTT memory; at clinic open time
- RTT obtains Attending MD approval if Pt IP/OP status changed, as applicable
- RTT communicates change of Pt IP/OP status to Admin, as applicable
- Admin flags in EPIC if Pt changes from OP to IP, as applicable
- EPIC transfers IP account to MQ, as applicable
- Admin creates Pt OP account in EPIC if Pt changes from IP to OP, as applicable
- Admin verifies Pt OP status by unchecking IP checkbox in MQ if Pt changes from IP to OP, as applicable
- RTT views Mq Tx record
- RTT schedules IP Pt transportation (at clinic open time) or contacts OP Pt
- Admin queues Pt to RN
- RN or CST takes vital signs (each day)
- RN administers pre-medications as applicable
- RN communicates with ancillary services as applicable
- RN documents assessment in EPIC
- RN queues Pt to Provider via page/verbal communication, if status check day
- RN queues Pt to RTT for Tx
- RTT perform site setup, per site, verifies setup via self-check, approves setup in Mq
- RTT walks Pt from recovery room to vault
- RTT verifies Pt ID by 2 independent means
- Pt confirms ID on monitor in maze
- RTT performs Pt site setup, according to setup notes (immobilization devices, shifts), per site
- RTT compares R&V setup (clinical parameters, Tx consent) vs. actual, with RTT partner via time-out
- RTT documents Tx setup details & RTT partner time-out agreement
- RTT images Pt per Attending's orders
- MD and PHYS reviews imaging for acceptable setup prior to beam on
- RTT deliver Tx per Attending's Rx
- RTT observes Pt on monitors for duration of Tx
- Mq records Tx
- RTT manually record treatment if Mosaiq connection fails
- Repeat from "RTT performs Pt site setup", if untreated sites remain
- RTT captures charge if Tx sucessful
- RTT queues Pt to next location, if Mq prompts that appt's remain
- Pt leaves vault
- RTT messages Attending for final Tx now, via Resident, if final Tx
- RTT marks Pt as completed & inactive, if final Tx
- MD orders post-Tx appointments, labs, imaging, special procedures from Admin via Onc Flowsheet, email, call, EPIC inbasket, if last status-check

On-Tx quality management

- PHY completes weekly physics chart check, including Continuing Physics charge captures
- MD, RN, Ancillary Services completes daily/weekly management visit (pre-medications), social work, and nutrition
- Admin reviews charges daily

Post-Tx completion

- Pt carries Yellow Sheet to Front Desk to trigger post-tx Appt scheduling
- Admin schedules post-Tx appointments using information from Onc Flowsheet
- PHYS performs final EOT chart check
